# American Dental Convention

**Published:** 1871-07

**Authors:** 


### ARTICLE IV.

### *American Dental Convention.*

The undersigned Committee of Arrangements would inform the *dental profession* that they have made arrangements for holding the seventeenth annual meeting of the American Dental Convention at Saratoga Springs, commencing on Wednesday the 9th day of August, at 11 A. M.

Should the attendance be as large as anticipated, a liberal deduction will be made to members of the convention by the proprietors of the two principal hotels, with the assurance that no effort will be spared to make their guests as comfortable as possible.

Arrangements will be made for the display and exhibition of dental materials and appliances of all kinds. Also for clinical operations, during the session.

Dentists and others desirous of exhibiting instruments, materials or improvements in any department of dentistry, are particularly invited to present them as early in the session as possible, and to notify the committee of their intention to do so.

As August is a very busy month with hotel keepers, it will be advisable for those intending to be present to notify the committee of their wishes &c., at 25 West 23rd St., New York.

Efforts are making to secure a reduction of fare by rail and steam boat.

J. G. Ambler, J. S. Lattimer, W. B. Hurd, J. S. Smith,
*Committee of Arrangements.*

---

